# Observations on Relapsing Fever as If Occurred in Philadelphia in 1869-70

**Published:** 1871-01

**Authors:** 


### Abridgments from our Exchanges.

---

Observations on Relapsing Fever as it Occurred in Philadelphia in 1869–70, is the title of a paper contributed to the *American Journal of Medical Sciences,* by Dr. J. S. Parry, of Philadelphia. The disease made its appearance in the early part of November, 1869. The first cases appeared in parts of the city filled with decaying garbage, traversed by dark and filthy alleys, and inhabited by people who lived, much crowded, in illy ventilated apartments. Every attempt to trace their origin has failed.

The disease is generally supposed to have its origin in want of food, although many authorities acknowledge over-crowding and typhoid poison as causes. At first the writer shared this view, but, with one exception, he found the persons effected to be well fed and some were even fat. The disorder did not occur among the "vagrant and unemployed," but heads of families afflicted held positions in stores and factories; hence, he was led to seek another cause for the malady.

He noticed that in nearly every case the patients were the subjects of over-crowding in their dwellings, and especially in their sleeping apartments; four or five persons often occupied a room 11 by 15 feet, and with little or no ventilation. To such an astonishing degree was this crowding and ill-ventilation found to exist that his only wonder was that a worse scourge had not long before attacked them. Exactly this element of bad hygiene he believes to be the cause of the epidemic.

Dr. Parry is of opinion that this disease is contagious, as nurses sleeping with patients were invariably attacked, while other members of the family were less liable to be effected; yet he says if apartments occupied by the sick were well ventilated, they could be visited with impunity, and patients taken to well ventilated hospitals were not liable to propogate the disease.

When a patient is confined in a close room, the disease may spread until all members of the family are affected and have to be taken to hospital for nursing.

The disease may be carried by fomites, as was verified in a number of cases.

All the sufferers, with two exceptions, were Irish or born of Irish parents.

No age was exempt; it attacked old and young alike, and there were twice as many women as men, a fact doubtless due to the fact that men are more exempt from the evils of over-crowding and contagion.

The period of incubation was from seven to fourteen days. The invasion was almost always sudden, the patient being able to fix the time of the attack with great precision.

In adults a chill was frequently the initial symptom, which was often accompanied or preceded by headache, giddiness, nausea, and vomiting, and in children profuse vomiting usually began the attack. The cold stage was rapidly followed by headache, high fever, restlessness, insomnia, pain in the back and limbs, with rapid pulse; the tongue was furred; there were epigastric pains, nausea, and vomiting. These all persisted until the fifth, seventh, or ninth day, when they suddenly stopped, to be repeated at the end of the second week of the disease.

Studying the symptoms separately, the face was noticed, after the fever set in, to be flushed—red or purple. There was no dullness or stupid expression, such as is common in continued fever.

During the remission, and after the second relapse, the face was frequently pale, puffy, or velvety in its look. At the onset of the febrile stage, the pupils were normal or rather contracted, and remained so until the crisis, when they became widely dilated, even in a well-lighted room; this condition continued three or four days, and then disappeared.

The skin was always very hot during the paroxysm and, until the month of February, it was found in all patients dry; then it often was seen to be bathed in perspiration; this, however, neither gave relief or reduced the temperature.

When the temperature and pulse fell, the perspiration was excessive. The sweats at the crisis were frequently attended by an eruption of sudamina all over the body.

During the primary paroxysm, the tongue was coated white or yellowish-white, to become, after three or four days, red at its tip and edges. During the intermission it partly cleaned off, and, in the relapse, was in different cases moist, or dry and cracked, and a part or the whole of the coating came off; but during the last of the epidemic these latter phenomena were not noticed.

There was usually anorexia during the paroxysms, the appetite returning in the intervals. The bowels were, as a rule, constipated, both during the attack and remission; in exceptional cases, however, there was a slight diarrhæa. In all the eases there was the epigastric tenderness, with nausea during both the primary paroxysm and relapses.

In all the cases, there was enlargement of the liver and spleen.

During the primary paroxysm, the headache was constant and intense; but delirium, stupor, and subsultus, so common in typhoid, were wanting.

Insomnia was constant, often in spite of all remedies against it.

Severe pains in the back and joints were almost always present in the paroxysms, and often during the intermission.

During the remission and convalescence, a loss of power in the extremities for a few days was frequently noted, and numbness was not unusual in the limbs.

More or less prostration always existed, but it was slight compared with that in typhus or typhoid.

The pulse always rose rapidly, reaching in the febrile stage always as high as 110, and often 130 or 140. It was highest just before the crisis, and fell as rapidly as it rose; it often then became abnormally slow.

The heat of body is remarkable; during the fever it reaches 104 or 106 deg. F.; during the intermission, it falls below the normal.

In all cases, the urine during the fever was scanty and high-colored.

In but few cases was there a second relapse, and by treatment, in most cases, the disease can, Dr. P. thinks, be arrested before a relapse occurs.

All the cases recovered, though in some instances very slowly.

Quinine nor other anti-periodics have any power to cut short this disease. It seems as though the therapeutics of relapsing fever is "almost entirely limited to the diminution of the body heat, prevention of complications, and the relief of a few special symptoms." In this epidemic, the symptoms most difficult of relief were the insomnia and pain; they resisted all remedies; it seemed almost impossible to get the patients to sleep, although bromides and opium were freely used. Purgatives, although given in large doses, acted ineffectually or not at all. No remedy was found that would prevent the relapse.

